# Intelligent Recommendation Model of Contemporary Pop Music Based on Knowledge Map

**DOI:** 10.1155/2022/1756585

**Published:** 2022-02-09

**Authors:** Yan Zhang

**Affiliations:** Department of Music, General Graduate School, Sejong University, Seoul 05006, Republic of Korea

## Abstract

With the advent of the era of big data, the rise of Web2.0 completely subverts the traditional Internet model and becomes the trend of today's information age. Simultaneously, massive amounts of data and information have infiltrated various Internet companies, resulting in an increase in the problem of information overload. In the online world, learning how to quickly and accurately select the parts we are interested in from a variety of data has become a hot topic. Intelligent music recommendation has become a current research hotspot in music services as a viable solution to the problem of information overload in the digital music field. On the basis of precedents, this paper examines the characteristics of music in a comprehensive and detailed manner. A knowledge graph-based intelligent recommendation algorithm for contemporary popular music is proposed. User-defined tags are described as the free genes of music in this paper, making it easier to analyze user behavior and tap into user interests. It has been confirmed that this algorithm's recommendation quality is relatively high, and it offers a new development path for improving the speed of searching for health information services.

## 1. Introduction

Recommendation system is a smart software technology that can provide personalized recommendation services for users based on their interests, preferences, and characteristics [1]. The amount of music available is growing at an exponential rate, making it difficult for users to search for and find related music. As a result, researchers from all over the world are increasingly focusing on music retrieval research [2]. Intelligent music recommendation has become a research hotspot in current music services [3] as an effective way to solve the problem of information overload in the field of digital music. As the number of users has grown significantly, more and larger amounts of data have been generated. The recommendation system was created to address the issue of how to best use these data in order to provide more accurate services. To retrieve eligible music benefits, traditional music retrieval uses metadata. This retrieval method necessitates users remembering relevant information from the target track prior to retrieval, which is incompatible with users' fast-paced lives [4]. In modern life, what users need is to be able to play music that suits their interests continuously. However, how to provide different users with a list of songs that suit their interests or recommend interesting songs for them has become a problem that the current music recommendation system needs to solve. Intelligent music recommendation system is faced with the problems of accuracy, diversity, and novelty of recommendation, while its data set is sparse and information is missing.

In recent years, although the recommendation algorithms [5] in the fields of movies, news, and books have emerged one after another, there are not many recommendation systems in the field of music, mainly because the data in this field are often not made public, and unlike the ratings we usually use, there is basically no public data set that mentions the user's ratings [6]. Intelligent music recommendation system is an effective way to solve “information overload.” The recommendation system mines users' potential interest preferences according to their historical behaviors, calculates the similarity between users and resources, and recommends music resources that users may be interested in [7]. At present, in the research of intelligent music resource recommendation methods, researchers focus on user feature modeling and resource attribute modeling and recommend by calculating the similarity between users and resources. In most cases, people background music and listen to music while studying, working, or exercising [8]. The emergence of music recommendation system enables users to quickly and continuously obtain music that suits their interests. The emergence and development of Web2.0 and social media allow users to act freely [9]. Users can define labels for music through their own understanding and feelings, and different labels can interpret users' understanding of music from different angles. Knowledge map is often used in intelligent search engines, recommendation systems, and other fields to improve its accuracy because of its strong organizational ability and relationship processing ability [10]. At present, the recommendation system based on a knowledge map is mainly divided into feature-based recommendation and path-based recommendation. Knowledge features are extracted through knowledge feature learning, and the ability of feature extraction, that is, the quality of knowledge representation, not only determines whether it can bring useful information for recommendation but also determines the quality of knowledge map construction. Therefore, this paper constructs a small-scale knowledge map in the field of machine learning and, on this basis, proposes a music resource recommendation algorithm based on a knowledge map, which integrates music connectivity and user interests.

In a broad sense, the recommendation system can be understood as recommending items that users may be interested in. The recommended items can be movies, music, books, short videos, and so on, depending on the specific field of application [11]. Today, with the continuous expansion of the Internet, the amount and types of information are increasing, and users put forward higher requirements for information retrieval and recommendation of related items [12]. Because different users have different interest preferences, the focus of attention, personal personality orientation, and so on, in order to meet the different recommendation needs of different users, an intelligent recommendation system featuring that everyone can get different recommendations from others came into being [13]. Traditional music recommendation algorithms based on “User-Item” usually only pay attention to the two-dimensional scoring relationship, ignoring the situation information of users when listening to music [14]. This paper proposes an improved algorithm based on a knowledge map, which is based on the traditional music recommendation algorithm and incorporates the users' situation information when listening to music. Simultaneously, this paper examines the existing music recommendation system in depth, combining it with some of the benefits provided by the recommendation algorithm. By analyzing the recommendation results, we can see that the system's overall performance is good and that it can effectively recommend results that users might be interested in to a degree, essentially achieving the system's intended goal.

## 2. Related Work

Literature [15] proposes to recommend music that meets users' needs and preferences by analyzing users' listening habits and the characteristics of the music itself. Literature [16] proposes that, in order to meet the personalized needs of different users for music, major well-known music recommendation systems are increasingly favored by consumers. Convolution matrix decomposition based on file additional information is proposed in literature [17]. Literature [18] introduces the information in the knowledge base such as structure information, text data, and image data into the recommendation system to improve the quality of the recommendation system. A session-based recurrent neural network recommendation model is proposed in literature [19]. A multirate depth learning model based on time recommendation is proposed in literature [20, 21]. Literature [22] introduces the concept of similarity based on meta path, in which meta path is a path composed of a series of relationships defined between different object types. A new similarity measure called PathSim is defined under the meta path framework, which can find objects in peer-to-peer networks. References [23,24] propose the method of using depth hierarchy model in film recommendation task. References [25, 26] propose a novel knowledge representation learning method named embodied type by using the naming of entity level type. Literature [27] introduces the potential features based on meta paths to represent the connectivity between users and projects on different types of paths and uses the relationship heterogeneity in the information network to provide high-quality intelligent recommendation results. Based on previous studies, this paper proposes an intelligent recommendation algorithm for contemporary pop music based on a knowledge map. The algorithm comprehensively analyzes user behavior by analyzing user interests and user preferences for different musical gene characteristics.

## 3. Methodology

### 3.1. Related Theories and Technologies

Music is an abstract art form that reflects people's real life and expresses their inner thoughts [28]. It is also a way to express their feelings and repose their feelings. With the rapid decline of the status of traditional records and the rapid promotion and spread of digital music, online audition and mobile music have become new ways for people to obtain music [29]. However, the keywords used by users do not correspond well with the item description tags, and converting audio information into digital information will lead to problems such as increased computation and prolonged response time. This content-based method also ignores the similar interests of different users, so it cannot adapt to the community-based network well.

Feature-based recommendation algorithms directly represent knowledge using a knowledge map, which fails to introduce multihop relationships and makes it difficult to use knowledge semantic network information. The path-based recommendation algorithm makes use of the knowledge map's multihop knowledge and effectively uses the knowledge map's semantic network information, but it usually relies on prior knowledge. As a result, studying the application of the knowledge map in the recommendation system is of great research value in addressing the problem of how to effectively use the semantic association information of knowledge in recommendation algorithms.

**Figure 1:**
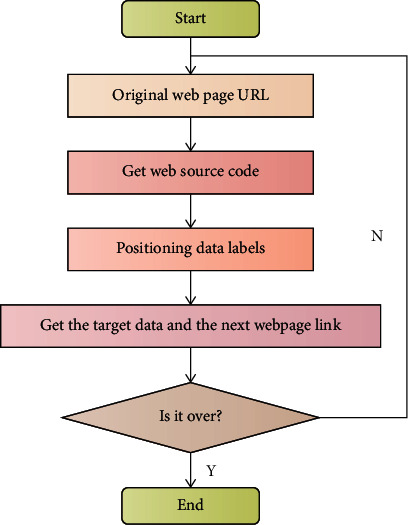
Process of obtaining web page data by crawler.

A web crawler is a program that requests websites and extracts information from them [30]. In the study of music recommendation systems, there are numerous data sets. However, it was discovered in this paper through the collection of various data sets that none of the existing data sets can contain weather information when users listen to music. This paper abandons existing public data sets in favor of using a web crawler to capture specific data sets in order to meet the research needs of intelligent music recommendation. The process of obtaining web page data by crawler is shown in Figure 1.

**Figure 2:**
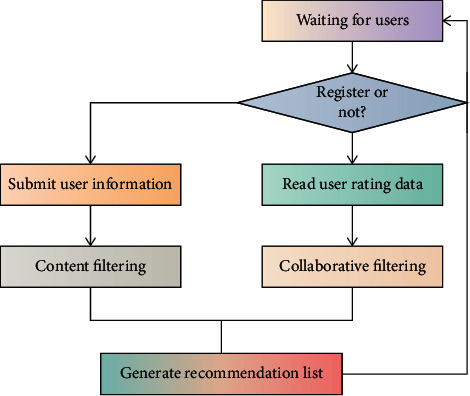
Technical framework of knowledge map.

The construction of the knowledge map mainly includes three processes. They are information extraction, knowledge fusion, and knowledge processing. Through the above process, we can build a relatively complete and reliable knowledge map. According to the actual situation of users, this paper adopts a recommendation algorithm based on the knowledge map to form a recommendation list.  Figure 2 shows the flowchart of intelligent recommendation of popular music based on the knowledge map.

Recommendation systems are intelligent software technologies that can provide personalized recommendations for users based on their interests, preferences, and characteristics. There are many definitions of recommendation systems available today, and different fields have different rules for defining them. The recommendation system is primarily used to estimate a user's preference for items that they have never seen before. It can be roughly divided into scoring prediction, ranking prediction, and classification prediction based on the output of the recommendation system. The scoring prediction is primarily aimed at the scoring matrix, with the goal of supplementing the matrix's vacancy scores. The ranking prediction is primarily intended to recommend items to a specific user who may be interested in them. Classification prediction is the process of categorizing items and recommending them to users who are interested in that category. Although the content-based recommendation is simple to calculate and understand and has been widely used in some fields, such as text recommendation, it is not without flaws that limit its development, as evidenced by the wide range of content feature extraction and recommendation results.

### 3.2. Intelligent Music Recommendation System Based on Knowledge Map

The algorithm approaches the target user's score of an item according to the score of the target user's nearest neighbor. Define the target user *a* and the item *i* that has not been rated excessively, and then predict the score of *a* to *i*:

(1) $$\begin{array}{c} p_{a,i}={\bar{r}}_{a}+\frac{\sum_{u=1}^{K}\left(r_{u,i}-{\bar{r}}_{u}\right)\omega_{a,u}}{\sum_{u=1}^{N}\omega_{a,u}}. \end{array}$$

Among them, *r*_*u*,*i*_ represents the score of user *u* on item *i*, *r*_*u*_ and ${\bar{r}}_{a}$ represent the average scores of user *a* and user *u*, respectively, and *ω*_*a*,*u*_ represents the similarity between user *u* and user *a*.

However, the collaborative filtering algorithm based on items thinks that users' scores of different items are similar. When users' scores of a certain item need to be estimated, users' scores of several similar items of the item can be used to estimate, as shown in the following formula:

(2) $$\begin{array}{c} p_{a,i}={\bar{r}}_{i}+\frac{\sum_{k=1}^{M}\left(r_{a,k}-{\bar{r}}_{k}\right)\omega_{i,k}}{\sum_{k=1}^{M}\omega_{i,k}}. \end{array}$$

Among them, ${\bar{r}}_{i}$ represents the average score of item *i*, and *ω*_*i*,*k*_ represents the similarity between item *i* and item *k*. In actual commercial applications, user-based collaborative filtering algorithms are more efficient than item-based. The number of songs in the corpus used in this paper is far greater than the number of users. In this paper, a user-based collaborative filtering algorithm is used as a comparative experiment to improve efficiency.

Knowledge map can filter data from the Internet and has multisource heterogeneous association information among entities. The filtered association information can fine-tune the feature information in users' projects, calculate the correlation between users and users, users and projects, and projects more precisely, and improve the recommendation system's interpretability, diversity, and accuracy.

If the current user's neighbors listen to a certain piece of music many times, it will greatly affect the recommendation effect. Therefore, in order to avoid this deviation, according to the characteristics of this corpus, the prediction score formula in collaborative filtering is treated as follows:

(3) $$\begin{array}{c} P_{a,i}=\frac{\sum_{u=1}^{K}\left(r_{u,i}-{\bar{r}}_{a}/\sigma_{a}-{\bar{r}}_{u}-{\bar{r}}_{a}/\sigma_{a}\right)*\omega_{a,u}}{\sum_{u=1}^{n}\omega_{a,u}}. \end{array}$$

The threshold *T*_*r*_ of the number of times of listening to the same songs between two users is selected, and the number *K* of similar users of the current user is selected. After many experiments, it has been proved that these two parameters are *T*_*r*_=20 and *K*=15, and the experimental effect is the best.

Knowledge features are extracted through knowledge feature learning, and the ability of feature extraction, that is, the quality of knowledge representation, not only determines whether it can bring useful information for recommendation but also determines the quality of knowledge map construction. When learning vectors, the knowledge model needs to construct negative triples to train the loss function. The usual method is to construct negative samples by randomly replacing the head entity or tail entity in the correct triples. The random method is effective at the beginning of training. However, with the continuous training, the scores of these randomly generated negative triples may exceed the sum of the correct triples scores and interval values, resulting in zero losses. As a result, the convergence speed gradually decreases, and the best result cannot be obtained.

For each relation *r* in the knowledge graph, two values are first counted. One is the average number of tail entities corresponding to each head entity, denoted as *N*_*tph*_. The other is the average number of head entities corresponding to each tail entity, denoted as *N*_*hpt*_. Then, defining the probability *p*, the calculation formula is as follows:

(4) $$\begin{array}{c} p=\frac{N_{tph}}{N_{tph}+N_{hpt}}. \end{array}$$

Then, the replacement head entity and replacement tail entity obey the Bernoulli distribution with parameter *P*. Set

(5) $$\begin{array}{c} X=\left\{\begin{array}{cc} 1, & \text{Replace head entity,}\\ 0, & \text{Replace tail entity.} \end{array}\right. \end{array}$$

Then the distribution law of *X* is

(6) $$\begin{array}{c} P\left\{X=x\right\}=p^{x}{\left(1-p\right)}^{1-x},\;x=0\cdot 1. \end{array}$$

The purpose of a content-based recommendation algorithm is to make recommendations for users based on the similarity of their interests and project contents and to make recommendations independently for each user without taking into account the interests and hobbies of other users. For text information recommendation, this method is simple and effective. The goal of learning is to encode entities and relationships into a continuous low-dimensional vector space, according to the knowledge map. Many existing methods focus solely on learning representations of structured information from triples, ignoring the rich hierarchical type information of entity species available from most knowledge maps.

Assuming that the set that user *u* and user *v* have scored together is denoted by *I*_*c*_ and *I*_*u*_, *I*_*v*_ are the set of items rated by user *u* and user *v*, respectively, the similarity sim (*u*, *v*) between user *u* and user *v* is

(7) $$\begin{array}{c} \text{sim}\left(u,v\right)=\frac{\sum_{i\in I_{c}}\left(R_{u,i}-{\bar{R}}_{u}\right)\left(R_{v,i}-{\bar{R}}_{v}\right)}{\sqrt{\sum_{j\in I_{u}}{\left(R_{u,j}-{\bar{R}}_{u}\right)}^{2}}\sqrt{\sum_{j\in I_{v}}{\left(R_{v,j}-{\bar{R}}_{v}\right)}^{2}}}. \end{array}$$

Among them, *R*_*u*,*i*_ represents the rating of user *u* on item *i*, and ${\bar{R}}_{u}$ and ${\bar{R}}_{v}$, respectively, represent the average rating of user *u* and user *v* on the item. *I*_*c*_ represents the set of items scored by users *u* and *v*, *I*_*u*_ represents the set of items scored by user *u*, and *I*_*v*_ represents the set of items scored by user *v*.

Assuming that the set that user *u* and user *v* have evaluated at the same time is represented by *I*_*c*_, the similarity sim(*u*, *v*) between user *u* and user *v* can be expressed by Pearson similarity as

(8) $$\begin{array}{c} \text{sim}\left(u,v\right)=\frac{\sum_{k=1}^{K}\left(R_{u,k}-{\bar{R}}_{u}\right)\left(R_{v,k}-{\bar{R}}_{j}\right)}{\sqrt{\sum_{k=1}^{K}{\left(R_{u,k}-{\bar{R}}_{u}\right)}^{2}}\sqrt{\sum_{k=1}^{K}{\left(R_{v,k}-{\bar{R}}_{j}\right)}^{2}}}, \end{array}$$

where *R*_*u*,*k*_ and *R*_*v*,*k*_, respectively, represent the ratings of user *u* and user *v* on item *k*, ${\bar{R}}_{u}$ and ${\bar{R}}_{v}$, respectively, represent the average ratings of user *u* and user *v* on a common rated item, and *K* is the number of repeated reviews of user *u* and user *v*.

Knowledge map is stored in triples composed of entities of entity relations, and entity relations will appear in multiple triples, presenting polysemy and complex relationship types.

In this section, several items with the highest analysis and forecast scores are presented to target users as recommended clearing units. User-based recommendation algorithm is more accurate when the data set is complete and the similarity algorithm is reliable, and it can avoid the differences in project content and make accurate recommendations and can implicitly and transparently explore the relationship between projects and user preferences.

## 4. Results Analysis and Discussion

The number of times users listen to a certain song to express the user's favorite degree of that song and the user's rating of the project according to his favorite degree is the data set used in this paper. When calculating the similarity between two documents in the context of data query, documents are frequently regarded as word frequency vectors, with the cosine value of the included angle between the two word frequency vectors used to express their similarity. The algorithm sort calculates the user-item relationship. A user query node in the algorithm is represented by a vector, with each dimension representing an item node in the association graph. Its value in this paper represents the number of times the user query node has listened to the music node or whether it has used the tag, indicating the degree to which users are interested in songs or tags.

**Figure 3:**
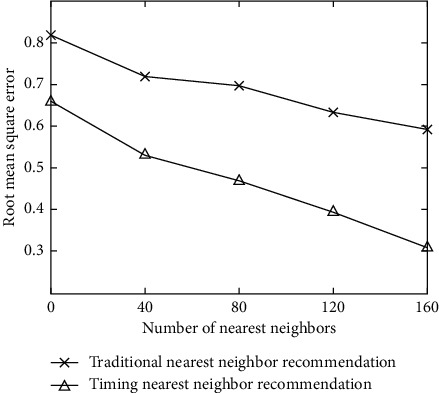
Comparison chart of root mean square error under different nearest neighbors.

The validity of the sequential user relationship model is the foundation of algorithm recommendation. To demonstrate the validity of the sequential user relationship model, the first step of the experiment is to compare the traditional nearest neighbor recommendation algorithm with the sequential nearest neighbor recommendation algorithm. The experimental results of the root mean square error comparison between them under different nearest neighbor numbers are shown in Figure 3.

From the graph, it can be seen that the effect of time-series nearest neighbor recommendation is always better than that of traditional nearest neighbor recommendation, whether the value of root mean square error is rising or falling, which proves the effectiveness of the time-series user relationship model. User preference extraction is the precondition of context awareness recommendation, and the purpose of contextual user preference extraction is to introduce contextual information into the user preference model. At present, there are two main types of situational user preference extraction technologies: quantitative analysis and qualitative analysis. The node type and relationship type in the knowledge map contain the design idea of the ontology library, and the entity information and semantic information between entities in the map show the extracted movie knowledge. The constructed knowledge map nodes and relationship types are reasonably designed, and the knowledge is accurate and comprehensive, which can be used for pop music retrieval.

**Figure 4:**
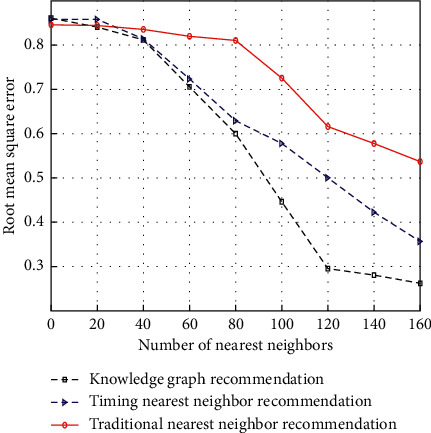
Comparison chart of root mean square error in different intervals.

In order to verify the effectiveness of the recommendation algorithm, the differences among traditional nearest neighbor recommendation, time-series nearest neighbor recommendation, and knowledge map recommendation are compared according to the different intervals in which users listen to music records. The root mean square error results of the three methods in different training set intervals are shown in Figure 4. The results fully prove the effectiveness of the recommendation.

**Figure 5:**
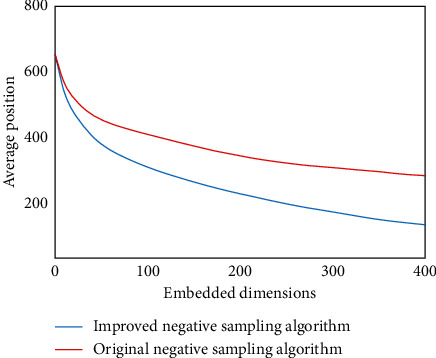
Average position under different embedding dimensions.

The matrix decomposition model, also known as the hidden semantic model, assumes that several hidden factors influence a user's rating of a specific item. The matrix decomposition model decomposes the large problem scale “user-item” scoring matrix into the product of two small matrices, the user potential feature vector matrix and the item potential feature vector matrix, respectively, and the product of the two matrices maps the user and the item to a common hidden factor space. We ran 20 tests for each combination configuration and then averaged the test results to find the best parameter configuration based on the average ranking of the correct triples.  Figure 5 shows the average ranking results under different embedded dimensions.

**Figure 6:**
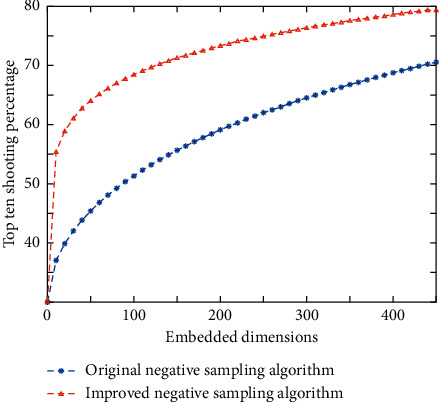
The top ten hit rates under different embedding dimensions.

From the figure, we can see that, in all the dimensions of the experimental test, the improved negative sampling algorithm has a significant improvement in the average ranking compared to the original negative sampling algorithm.  Figure 6 shows the top ten hit rate results in different dimensions.

**Figure 7:**
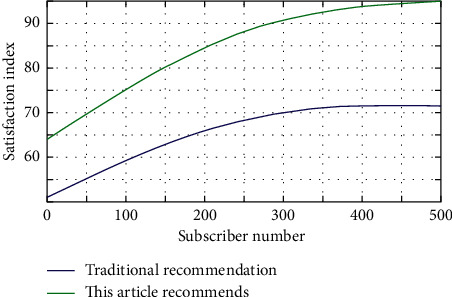
Comparison of user satisfaction evaluation.

The top ten hit rate results of negative sampling using the improved negative sampling algorithm are better than the original results in all dimensions, according to experimental results. The situational user preferences are converted into numerical scores in quantitative form using the quantitative analysis method, and mathematical calculations are performed using the corresponding preference extraction technology. In order to quantify situational user preferences, multidimensional vector scoring models and hierarchical models are commonly used. Heuristic and model-based technologies are two types of quantitative analysis methods. Similarity calculation, nearest neighbor algorithm, clustering, and other heuristic methods for extracting user preferences in scenarios are common. Users' playing records will be written into the database when they play music. All of the played music analyzed in this paper is recorded music for the sake of research. After existing users log in, the system will generate a list of recommendations based on the user's situation and then finish the prediction recommendation work. The user satisfaction of the music recommendation prototype system is assessed in this section, as well as the system's usability. The comparison of user satisfaction evaluation is shown in Figure 7.

**Figure 8:**
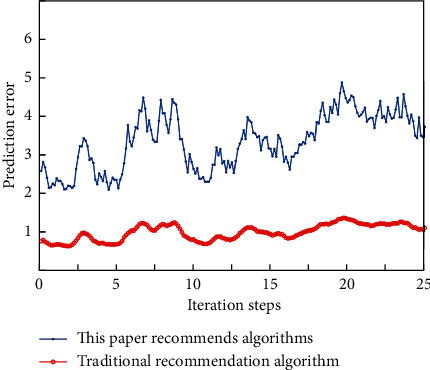
Comparison of two recommendation algorithms.

By evaluating the recommendation results, we find that the overall performance of the system is good, and it can effectively recommend the results that users may be interested in to a certain extent, basically reaching the expected goal of the system design. Comparative experiments are carried out for different algorithms, in which each group of experiments is designed to compare the recommended algorithm with a simple model after adding tag information. The comparison results are shown in Figure 8.

It can be seen that, in the same data set, the recommendation effect of the recommendation algorithm proposed in this chapter is obviously better than the collaborative filtering algorithm based on users. The purpose of the algorithm is to get the music items that are most closely related to the user. The content-based recommendation method has the advantages of avoiding cold start and data sparseness because it does not need other users' related data. However, the premise of this method is to extract the project content into meaningful features, and the features require good structure, which is very restrictive in many recommendation systems. An important part of the recommendation algorithm experiment is to select and set reasonable experimental standards. If we want to detect the performance of the algorithm well, we must have a reliable evaluation standard. With the reliable evaluation standard, we can also detect the areas to be revised in the recommendation algorithm.

The recommendation system based on the knowledge map constructed in this paper realizes the function of recommending popular music for users and shows the actual effect of the system. Experiments show that the algorithm can make full use of historical preferences and knowledge map relationship structure to deeply mine users' interests and hobbies. It has certain practicability.

## 5. Conclusions

With the advancement of the Internet and mobile networks, music has become an increasingly important form of entertainment in people's lives. Users, on the other hand, have higher standards for intelligent music recommendation. As a new application in the field of recommendation systems, intelligent music recommendation has a lot of research potential. Users' interest and loyalty to the system can be improved by using a knowledge map to analyze users' interests and accurately recommend personalized items that meet their interests, which is an inevitable trend in the development of commercial music systems and a hot field that scholars are scrambling to study. This paper builds a knowledge map-based recommendation system that is oriented to the application of music recommendation, realizes the function of recommending popular music to users, and shows the system's actual effect. A content recommendation algorithm with a unified embedding of behavior and knowledge features is proposed to solve the problem of knowledge map application in recommendation systems. To fully explore users' interests and hobbies, the algorithm makes extensive use of historical preferences and the knowledge map relationship structure. This paper uses the click prediction experiment to demonstrate the model's ability to dynamically learn related information and mine preferences deeply.

## Data Availability

The data used to support the findings of this study are included within the article.
